# Supplementary material for: Strong optical anisotropy in one-dimensional phosphorus wavy tubes
Source: Nat Commun. 2026 Feb 28;17:3286. doi: 10.1038/s41467-026-70129-4 (PMC13066532; doi:10.1038/s41467-026-70129-4)
Supplement: Supplementary file 1 — Supplementary Information [file 41467_2026_70129_MOESM1_ESM.pdf]

Supplementary Information

Strong optical anisotropy in one-dimensional phosphorus wavy tubes

Table of contents

Section 1. Discussion of wavy-tube phosphorus crystal synthesis 3

Section 2. Additional information on single-crystal X-ray diffraction -resolved structures .10

Section 3. Allotrope structure information comparison...12

Section 4. Additional information for energy dispersive spectroscopy mapping images of wavy-tube phosphorus crystal cross-sections 16

Section 5. Ellipsometry and optical constant extraction..17

Section 6. Discussion of theoretically calculated optical parameter....................................19

Section 7. Transmitted image of cross-polarized light 24

Section 8. Polar plot of Raman spectroscopy 25

Section 9. Additional information for second-harmonic generation spectra 27

Section 10. Energy band structure information obtained from ultraviolet photoelectron spectroscopy...29

Section 1. Discussion of wavy-tube phosphorus crystal synthesis

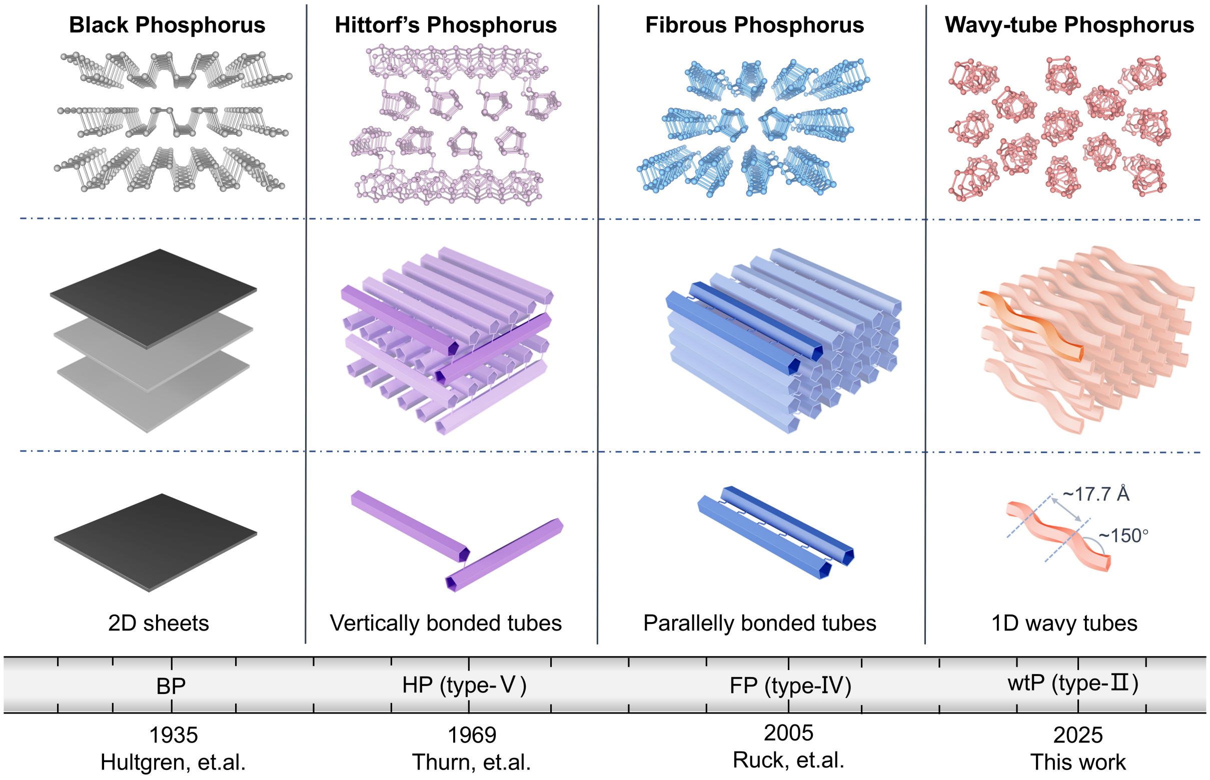

Supplementary Fig. 1 │ Elemental phosphorus allotropes. Crystal structures of elemental phosphorus allotropes and the corresponding investigators.

Elemental phosphorus materials have attracted much attention due to their various crystal structures and unique optoelectronic properties. One of the most famous elemental phosphorus allotropes is black phosphorus, first discovered by Bridgman in 1914[1], whose crystal structure was first reported by Hultgren, Gingrich & Warren in 1935 on the basis of powder diffraction data[2]. Its unique puckered structure confers photoelectric/electronic anisotropy, high carrier mobility, and direct bandgap[3]. According to the thermal analysis temperature and X-ray diffraction data of phosphorus allotropes discovered by Roth et al. in 1947, the red phosphorus allotropes were identified as type-I, type-II, type-III, type-IV, and type-V[4]. Among them, type-V red phosphorus was discovered as early as 1865 by Hittorf from molten lead and named Hittorf's phosphorus or

violet phosphorus (HP or VP)[5]. That was followed by the definition of the crystal structure of Hittorf's phosphorus in 1969 by Thurn et al., which was further defined by Zhang et al., who synthesized single crystals of Hittorf's phosphorus in 2020[6,7]. The vertically cross-linked tubes of Hittorf's phosphorus formed a unique double-layered sheet structure. Conversely, the parallelly cross-linked tubes created quasi-one-dimensional van der Waals crystals named fibrous phosphorus (FP)[8]. Based on the structure, Du et al. revealed the optical anisotropy of quasi-one-dimensional FP[9]. The investigations on phosphorus-based structures and properties not only advanced fundamental science but also significantly influenced the practical utilization of novel materials in various fields such as electronic devices, energy storage, and medical devices[10-14]. Theoretical predictions suggest that Type-II red phosphorus (Type-II RP) with a wavy tube structure may achieve strong anisotropy by breaking inter-tube symmetry, but its precise atomic configuration is unresolved, and there is also a lack of single-crystal materials verification[15-17]. Consequently, this study aims to synthesize elemental phosphorus crystals with one-dimensional motifs to determine the anisotropic properties.

Type-II red phosphorus represents a significant crystalline allotrope of elemental phosphorus. Since its initial discovery by Roth et al. in 1947[4], the determination of its precise crystal structure has remained a persistent and formidable challenge in the field. The core obstacle has been the extreme difficulty in obtaining high-quality single crystals suitable for atomic-resolution structural analysis. Early studies were severely hampered by the poor crystallinity of the initially available Type-II red phosphorus samples. Characterization relied primarily on techniques such as X-ray diffraction (XRD), Raman spectroscopy, and transmission electron microscopy (TEM). While these methods provided limited crystallographic parameters, they were insufficient for resolving the atomic-scale structure. The ambiguity surrounding Type-II red phosphorus during this period

is underscored by the widely cited yet data-limited JCPDS card (#00-044-0906) compiled in 1979, whose original source publication was seldom referenced.

Research progress stagnated for decades, with the importance of Type-II red phosphorus remaining obscured. A significant resurgence occurred in 2009 when Winchester et al. successfully re-synthesized Type-II red phosphorus via annealing amorphous red phosphorus under an inert atmosphere[15]. This reignited interest, and advances in characterization techniques over the past decade, particularly three-dimensional electron diffraction (3DED) and scanning transmission electron microscopy (STEM), have yielded substantial progress. These studies unequivocally established Type-II red phosphorus's unique structure, distinct from Type-IV (fibrous phosphorus, FRP), Type-V (violet phosphorus, VP), and known phosphorus nanorod variants. Landmark work by Yoon et al. and Zhang et al.[17,18] utilized advanced 3DED to determine the large triclinic unit cell parameters ($a \approx 9.230$ Å, $b \approx 9.128$ Å, $c \approx 21.893$ Å, $\alpha \approx 96.13°$, $\beta \approx 98.30°$, $\gamma \approx 102.44°$) and provided key insights into possible structural subunits like [P8]P2 and [P9]P2.

Despite these advances, the ultimate goal of determining the precise atomic coordinates and bonding configuration via definitive methods like single-crystal X-ray diffraction (SCXRD) remained elusive. This was primarily due to the persistent challenge of synthesizing high-crystallinity, defect-free, bulk single crystals. Crystals grown via various methods, including vapor-phase and solution-phase routes, consistently suffered from entanglement, disorder, and insufficient size/quality[15,19]. This low crystallinity fundamentally prevented atomic-scale structural resolution using SCXRD. While 3DED offered valuable clues, it could not fully replace the need for high-quality single crystals to achieve a complete and unambiguous structure model. This structural ambiguity has even led to lingering misconceptions within the phosphorus allotrope

community.

Our optimized chemical vapor transport (CVT) methodology successfully synthesizes millimeter-scale high-quality Type-II red phosphorus single crystals, overcoming a decades-long synthesis bottleneck through the following synergistic innovations: Precision Thermal Control—Thermogravimetric analysis identified 450°C as the critical decomposition threshold for Type-II RP. We established a temperature-dependent phase-selective relationship: temperatures >450°C yield violet phosphorus (VP) as the dominant phase, while lower temperatures limit crystal dimensions. Thus, growth was strictly maintained at the phase-transition threshold (450±5°C). Mineralizer Revolution—We replaced conventional iodine-based mineralizers (which form P-I bonds that impede crystal growth) with a novel Sn-based system. This eliminates parasitic reactions while optimizing phosphorus utilization efficiency. Kinetic Regulation—A low-pressure environment coupled with low-rate transport kinetics extended growth cycles to one week, enabling controlled crystal enlargement. Precursor Engineering—High-purity amorphous red phosphorus replaced instability-prone white phosphorus precursors, significantly enhancing reaction stability. This multiparameter optimization strategy fundamentally resolves historical challenges—including phase impurity, micron-scale crystallization, and byproduct interference—yielding macroscopic single crystals suitable for single-crystal X-ray diffraction analysis.

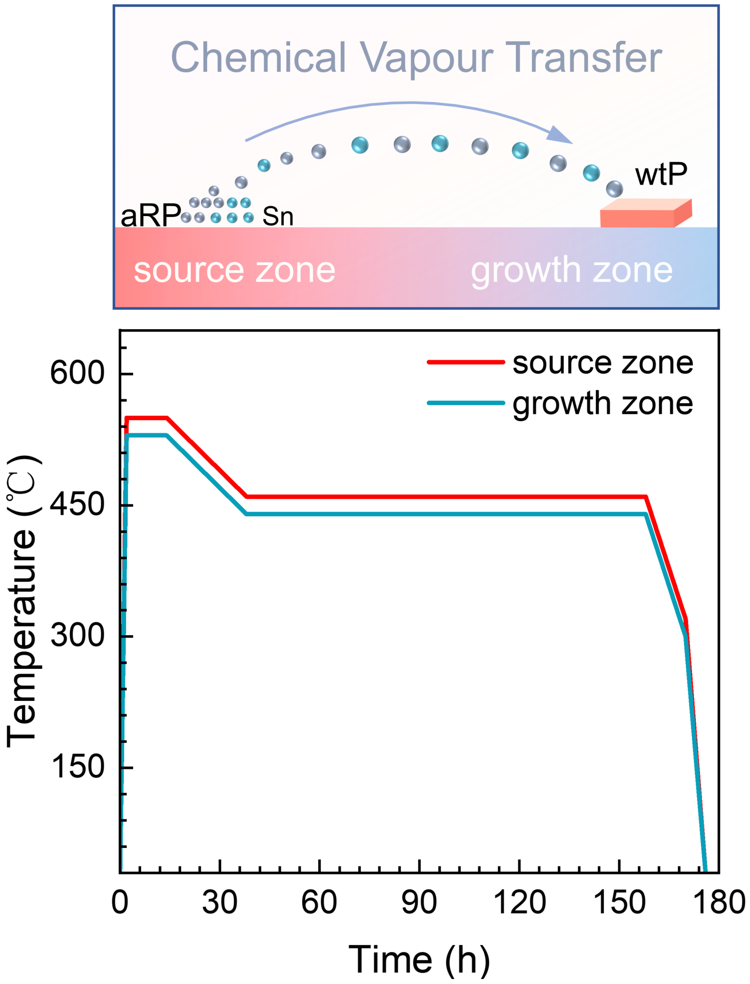

Supplementary Fig. 2 │ Schematic of synthesis of wavy-tube phosphorus crystal by chemical vapour transfer. Schematic diagram of chemical vapour transfer method and temperature-time programming of the dual-zone tube furnace for the synthesis of wavy-tube phosphorus (wtP). The temperature in the high-temperature zone (red line, source zone) is maintained at 20°C higher than that in the low-temperature zone (blue line, growth zone) throughout the growth. This temperature differential is crucial to the sublimation of precursors in the source zone and subsequent flow into the growth zone.

(a)

(b)

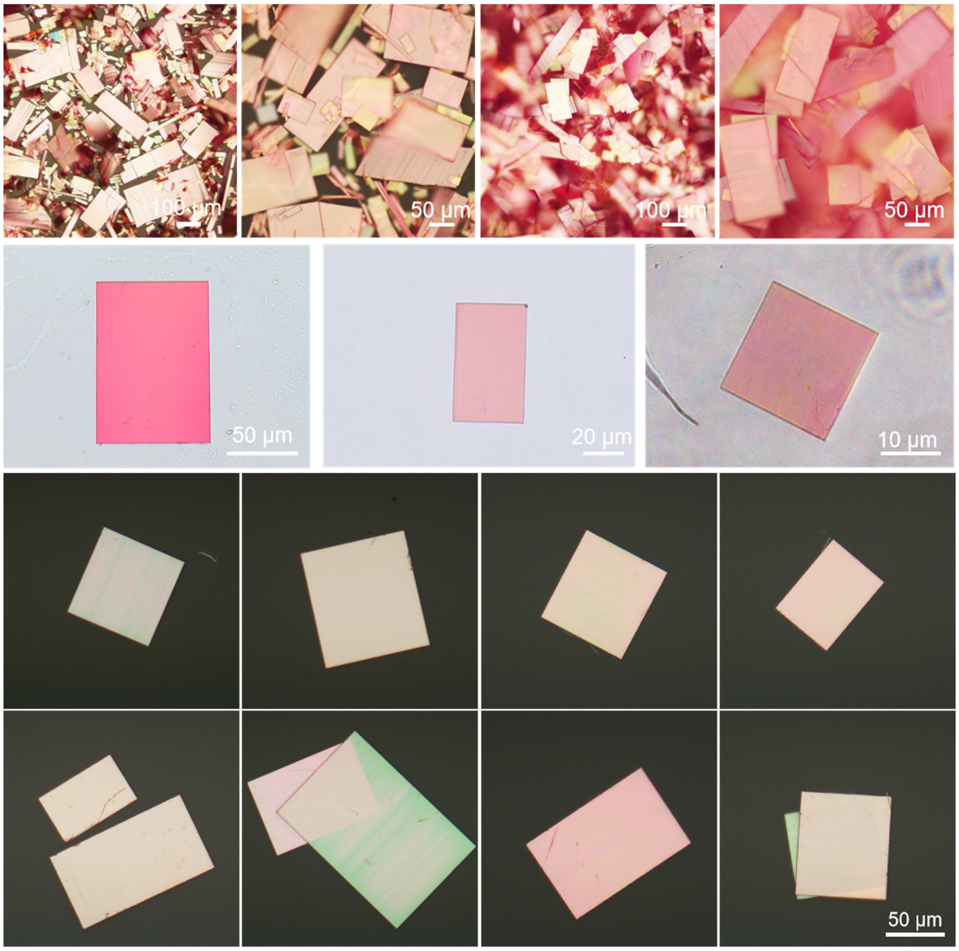

Supplementary Fig. 3 │ Optical photographs of wavy-tube phosphorus single crystals. (a) Optical photograph of the wavy-tube phosphorus **(**wtP) crystals under a bright field. (b) Optical photograph of wtP single crystals under a dark field. The edge lengths of wtP single crystals are ranging from 20 μm to 300 μm. White light balance calibration is conducted before taking the photographs.

Supplementary Table 1 │ Summary of reported synthesis parameters for vapor-phase synthesis of Type-II phosphorus

**Synthesis Method**	**Precursors**	**Container (Diameter×Length)**	**Temperature Schedule**	**Morphology**	**Sample Size**	**Ref.**
Vapor-phase method	a-RP (4 g)	Φ8~11× 150~450 mm	425~550 °C (Hot end)→380~545 °C (Cold end)	/	/	Roth, 1947[14]
Vacuum annealing	white phosphorus	Φ17×150 mm	420 or 436 °C for 16~24 h and cool to 300 °C for 12 h.	/	/	Rubenstein, 1966[20]
Vacuum annealing	white phosphorus	/	300 °C for 65 h, and then 450 °C for 16 h	/	/	Nechaeva, 1979[21]
Vacuum annealing	white phosphorus (0.06 g)	Φ8×40~50 mm	440 °C for 18 h	Nanorods	~1.3 μm (length)	Winchester, 2009[15]
Vacuum annealing	a-RP (400 mg)	Φ8×140 mm	466 °C for 16 h and cool to 300 °C for 12h.	Nanorods	~1 μm (length)	Yan, 2024[19]
Solvothermal reactions	a-RP (2g) + 1,2-diaminopropane	100 ml Teflon-line autoclave	170~230°C for 12 h	Platelets	300~500 nm (lateral size); 40~70 nm (thickness)	Yan, 2022[16]
Solvothermal reactions	RP + *N*-ethyl-1,2-ethanediamine	25 ml Teflon-line autoclave	250°C for 20 h	Nanosheets	~200 nm (lateral size); ~15 nm (thickness)	Duan, 2025[22]
CVT	RP (1.0 g) + I_2 (0 or 0.1 mg/mL)	Φ17.5×155 mm	400 °C→350 °C or 300 °C→250 °C	/	/	Schäfer, 1972[21]
CVT	a-RP (100 mg) + I_2 (100 mg) + Sn-coated SiO_2/Si wafer	/	600°C for 0.5 h, cool to 500°C in 5h, and maintain 1 h, cool to 150°C in 8 h	Nanowires	~10 μm (length); 200 nm (diameter)	Kim, 2023[17]
CVT	a-RP (400 mg) + I_2 (20 mg)	Φ8×140 mm	421~376 °C (hot end) with ~ 45 °C temperature gradient to cold end for 16 h	Thin films	/	Yan, 2024[19]
CVT	a-RP: Sn =10:1	Φ18×100 mm	550 ℃ cool to 460 ℃ in 25h, keep at 460 ℃ for 120 h (hot end), with 20°C different (cool end)	microplate	~100 μm (length); 10 μm (thickness)	This work

Section 2. Additional information on single-crystal X-ray diffraction -resolved structures

Supplementary Table 2 │ Crystal data of 1D wavy-tube phosphorus (wtP) single crystals.

Formula	wavy-tube phosphorus		
Temperature (K)	293	Z	6
Crystal system	Monoclinic	μ (mm^{-1})	17.484
Space group	$P2_1$	R_1, $wR_2[I>2\sigma(I)]$	R_1 = 0.0858
	a = 13.0518(3)		wR_2 = 0.2801
Unit cell dimensions	b = 34.4922(4)	F(000)	5400
	c = 18.8538(4)	Radiation	Mo K_α(λ=0.71073)
	β = 109.737(3)	Density (g cm^{-3})	2.317
Unit Cell volume	7989.1(3)		

$$R_1 = \sum\left||F_o| - |F_c|\right| / \sum|F_o|, wR_2 = \left\{\sum[w(|F_o|^2 - |F_c|^2)]/\sum\left[w\left|F_o{}^2\right|^2\right]\right\}^{1/2}$$

Regarding the PLAT029_ALERT_3_A for _diffrn_measured_fraction_theta_full in the Crystallographic Data (CCDC-2322552): Ideally (and a requirement for publication in Acta Crystallographica), the fraction should be close to 1.0 for theta-full greater or equal to sin(theta/lambda) = 0.6 (i.e. 25.24 degrees for MoKα and 67.7 degrees for CuKα radiation). In our crystallographic dataset, θmax = 76.45° (sin(theta/lambda) = 0.79) yields a diffrn_measured_

fraction of only 0.754, triggering a PLAT029_ALERT_3_A warning.

Different from ordinary three-dimensional bulk crystals, the test crystal wtP features one-dimensional chain structure, which exhibits high susceptibility to cleavage along both transverse and longitudinal crystallographic orientations, pseudo-defect planar twin boundaries form within the bulk crystal.

Consequently, only partial lattice reflections were usable during data reduction. Despite applying the SHELX truncation command (SHEL 999 0.79) to exclude high-angle reflections, the fraction remains suboptimal (0.754). This limitation originates from inherent diffraction characteristics of the crystal itself, rather than deficiencies in our data collection procedure.Three potential systemic errors could generally cause such alerts:

(1) A missing cusp of data due to data collection by rotation around the spindle axis only;

(2) Exclusion of intense reflections by the DENZO image processing package;

(3) Overestimation of crystal symmetry leading to incomplete scanning protocols.

In our experimental workflow, these error sources were systematically eliminated: Diffraction data were collected via ω-scan mode with full-sphere coverage using a Rigaku four-circle diffractometer, while data reduction and Structure refinement was performed with CrysAlisPro and OLEX2, respectively. Thus, we attribute the PLAT029_ALERT_3_A exclusively to intrinsic crystal properties (e.g., anisotropic diffraction or lattice imperfections), which do not compromise the structural solution.

Section 3. Allotrope structure information comparison

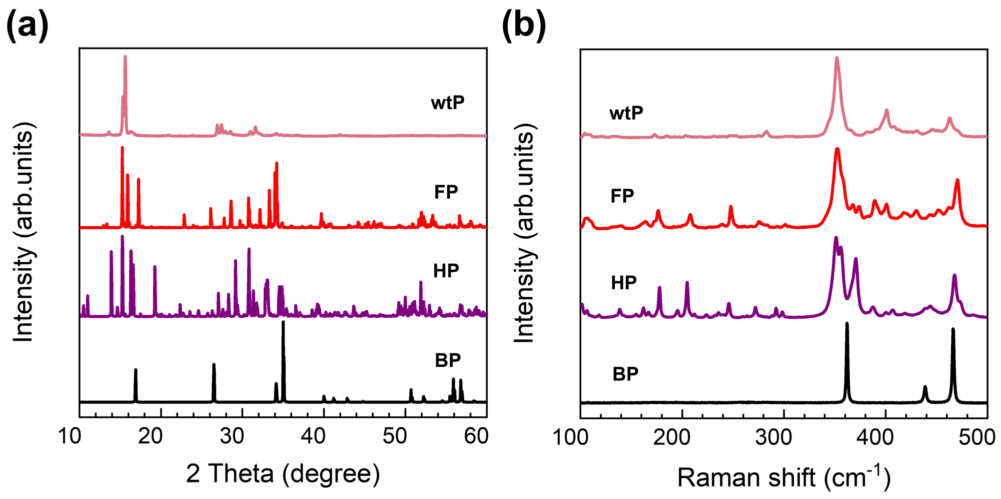

Supplementary Fig. 4 | X-ray diffraction patterns and Raman spectra of elemental phosphorus allotropes. (a) X-ray diffraction (XRD) patterns of wavy-tube phosphorus (wtP) single crystals with the preferred orientation and standard stimulated XRD patterns of FP, HP, and BP. (b) Experimental Raman spectra of wtP single crystals and FP, HP, and BP.

The XRD pattern of wtP shows three salient peaks at 13.6°, 15.6°, and 27.3°, which are different from those of the standard PDF cards of FP (ICSD-391323)[23], HP (ICSD-131503)[6] and BP (ICSD-23836)[24]. The Raman spectrum of wtP exhibits peaks at 355, 401, and 463 cm^{-1}. The Raman spectra of FP, HP, and BP are consistent with previous reports[25-29].

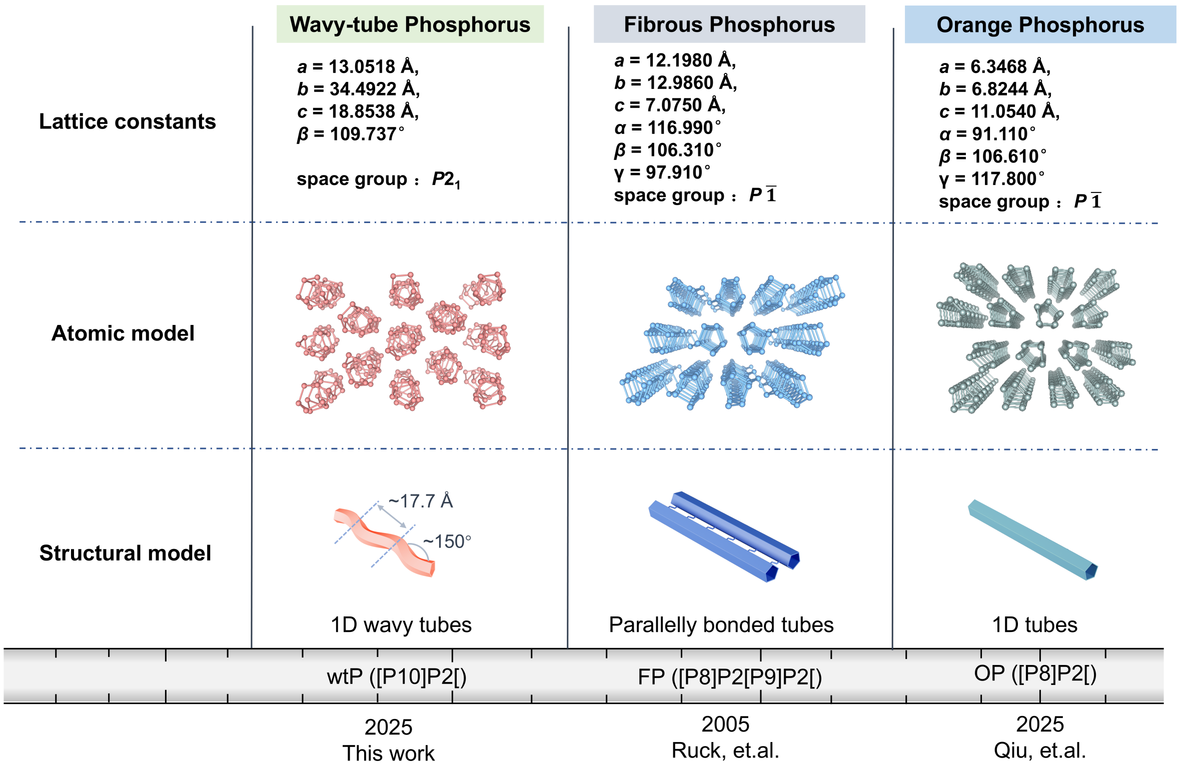

Supplementary Fig. 5 | Crystal structures of elemental phosphorus allotropes with tube unit. Lattice constants, atomic models, unit structure models of wavy-tube phosphorus (Type-II phosphorus), fibrous phosphorus, orange phosphorus.

As shown in Figure S5, a comparative structural analysis was conducted for three elemental phosphorus allotropes featuring parallel-chain motifs: fibrous phosphorus (FP)[8], the recently reported orange phosphorus (OP)[18], and the wavy-tube phosphorus (wtP) presented in this work. Both FP and OP exhibit relatively simple linear chain structures (composed of [P8]P2 units) and crystallize in the triclinic $P\bar{1}$ space group, whereas wtP is fundamentally distinct. It adopts a monoclinic $P2_1$ space group and displays a unique corrugated tubular topology—where each one-dimensional polygonal tube consists of 30 phosphorus atoms and possesses a characteristic wavy periodicity of 17.7 Å. This structural evolution from the simple linear chains of FP/OP to the complex wavy tubular network of wtP clearly demonstrates that wtP represents a previously unrecognized structural paradigm among elemental phosphorus allotropes.

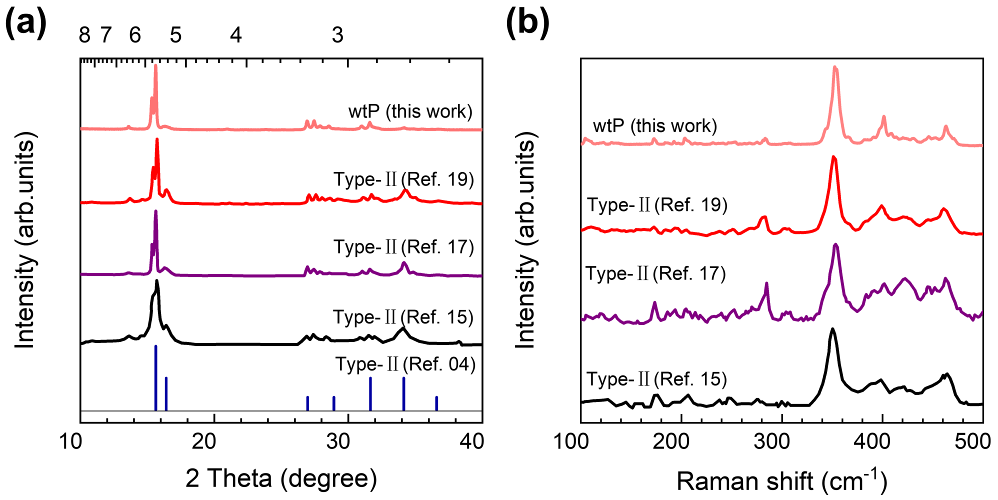

Supplementary Fig. 6 | Powder X-ray diffraction patterns and Raman spectra of Type-II red phosphorus. (a) Powder X-ray diffraction (PXRD) pattern of the wavy-tube phosphorus (wtP) powder without preferred orientation compared with PXRD patterns of type-II red phosphorus in literature [4,15,17,19]. (b) Raman scattering spectra of wtP crystals in this work and previously reported type-II red phosphorus. The peaks in the XRD patterns and Raman spectra of the as-prepared wtP are consistent with those reported for type-II red phosphorus. Meanwhile, the Raman spectra show less noise than those in the literature, suggesting that our sample has higher crystal quality.

Supplementary Table 3 │ Comparison of the typical Powder X-ray diffraction (PXRD) peaks between wavy-tube phosphorus (wtP) crystals and type-II red phosphorus first obtained by Roth et al[4].

**This work**		**Previous results**	
**D-spacing (Å)**	**Intensity**	**D-spacing (Å)**	**Intensity**
5.67	strong	5.66	strong
5.39	medium weak	5.39	medium weak
3.31	very weak	3.30	very weak
3.08	very weak	3.08	very weak
2.83	medium weak	2.82	medium weak
2.62	medium weak	2.62	medium weak
2.43	very weak	2.45	very weak

The wtP crystals have a similar color as type-II red phosphorus reported by Shaffer[15], which is different from the colors of FP, HP and BP[13,30]. The PXRD pattern of wtP shows the strongest diffraction peak for the 5.67 Å lattice spacing, and the other peak positions and intensities are consistent with Roth's results (Table S3)[4,15-17].

Section 4. Additional information for energy dispersive spectroscopy mapping images of wavy-tube phosphorus crystal cross-sections

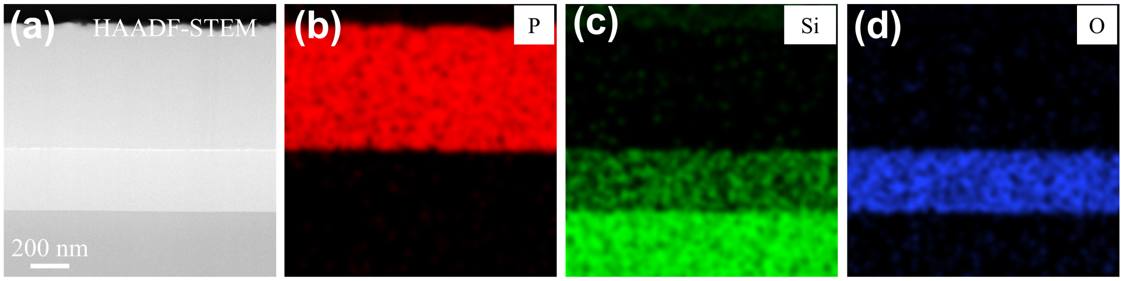

Supplementary Fig. 7 │Energy dispersive spectroscopy mapping images of wavy-tube phosphorus. (a) High-angle annular dark field-scanning transmission electron microscopy (HAADF-STEM) images of cross sections of wavy-tube phosphorus (wtP) single crystals and (b-d) Elemental maps.

Section 5. Ellipsometry and optical constant extraction

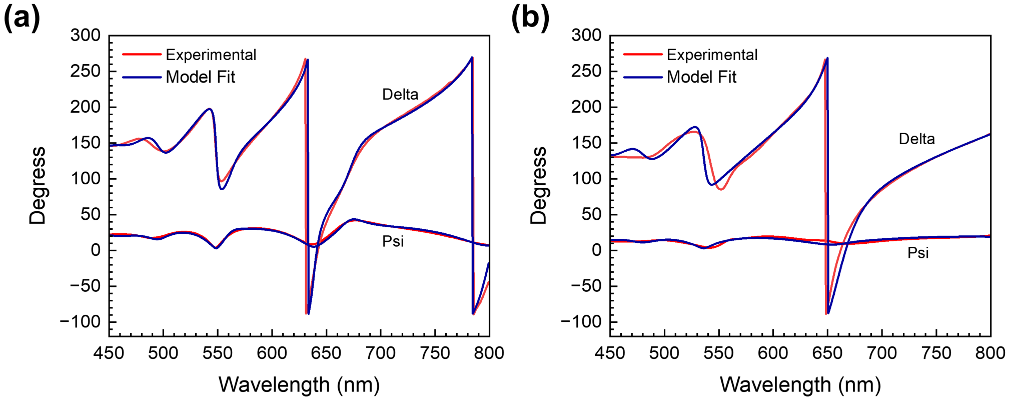

Supplementary Fig. 8 │ Optical constants extraction for wavy-tube phosphorus. Ellipsometry data and its fitting result in incident plane parallel to (a) and perpendicular to (b) *c**-axis.

For determination of the optical constants of the wavy-tube phosphorus (wtP), a wtP single crystal sheet was dry-transferred onto a quartz substrate. The ellipsometric data were acquired using a dual-rotator compensator ellipsometer (ME-Mapping-40F ellipsometer, Wuhan Eoptics Technology Co., Wuhan, China). The instrument featured a spot size of 40×60 µm with an incident angle of 64.95° (relative to the surface normal). Data analysis was performed using Eometrics software. The wtP sample was measured twice on the (20-1) crystal plane at two different directions parallel and perpendicular to the *c**-axis. The spectroscopic range for ellipsometry measurements spanned 280-1000 nm, though the optical parameter fitting range was restricted to 450-800 nm due to the thickness of the wtP single crystal. Following standard ellipsometer measurement protocols, spectroscopic ellipsometric data were presented as Psi and Delta values. An optical model of the quartz substrate and wtP structure was established within the Eometrics analysis software to generate corresponding simulated Psi/Delta polarization spectra (Fig. S8). The model was fitted using Tauc-Lorentz oscillators to match the measured polarization spectra,

specifying optical constant parameters for the material parallel and perpendicular to the c^*-axis, thereby extracting optical information such as refractive index and extinction coefficient.

Section 6. Discussion of theoretically calculated optical parameter

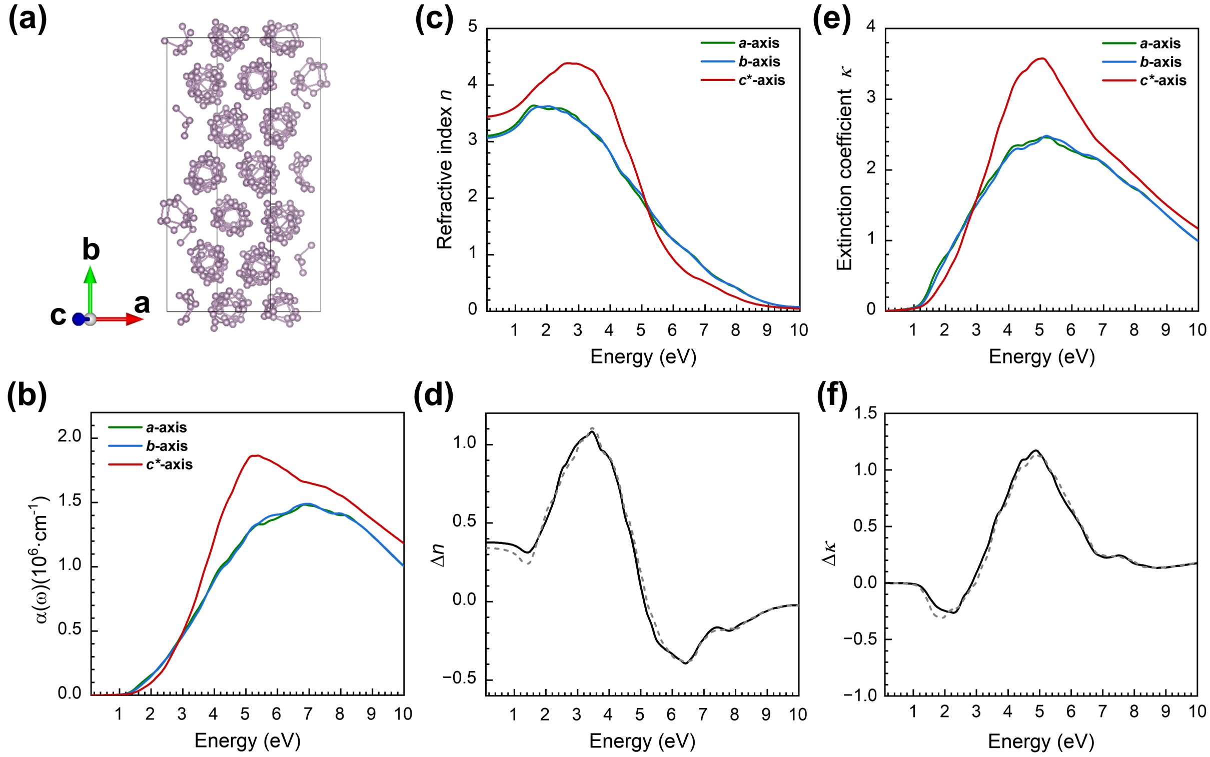

Supplementary Fig. 9 │ Theoretical optical properties of wavy-tube phosphorus crystal. (a) Calculation model, (b) absorption spectrum, (c) refractive index (n), and (e) extinction coefficient (κ) calculated along the a, b, and c^*-axis of the crystal lattice. (d) Birefringence (Δn) of the a-c^* (black dashed line) and b-c^* (solid line) planes. (f) Dichroism ($\Delta\kappa$) of the a-c^* (black dashed line) and b-c^* (solid line) planes.

As shown in Fig. S9b, its absorption spectrum exhibits distinctive anisotropy in the low-energy region (1-2.9 eV), where optical transitions dominated by electronic states arising from inter-tube van der Waals interactions cause absorption in the *a/b*-axis to be slightly higher than in the *c**-axis. However, when the photon energy exceeds 2.9 eV, electronic transitions exciting covalent bonds within the tubes become predominant. Consequently, the absorption intensity in the *c**-axis reverses and substantially surpasses that in the *a/b*-axis, forming a strong intrinsic absorption peak near 4.8 eV. The remaining optical responses are directly governed with the

intense absorption anisotropy, as illustrated in Fig. S9c where the refractive index along the *c**-axis exceeds that of the *a/b*-axis between 1-5 eV. Moreover, this substantial disparity culminates at ~3.54 eV preceding the strong absorption peak, inducing a birefringence of up to 1.09 in deep ultraviolet region (Fig. S9d). Correspondingly, dichroism show a theoretical peak value of 1.17 at 4.8 eV (Fig. S9f). Moreover, since the wtP crystal space group is $P2_1$ (monoclinic), its theoretically calculated optical properties clearly reveal biaxial optical characteristics. Its significant anisotropy is prevalent across the crystal planes containing the *c**-axis (Fig. S9d, f).

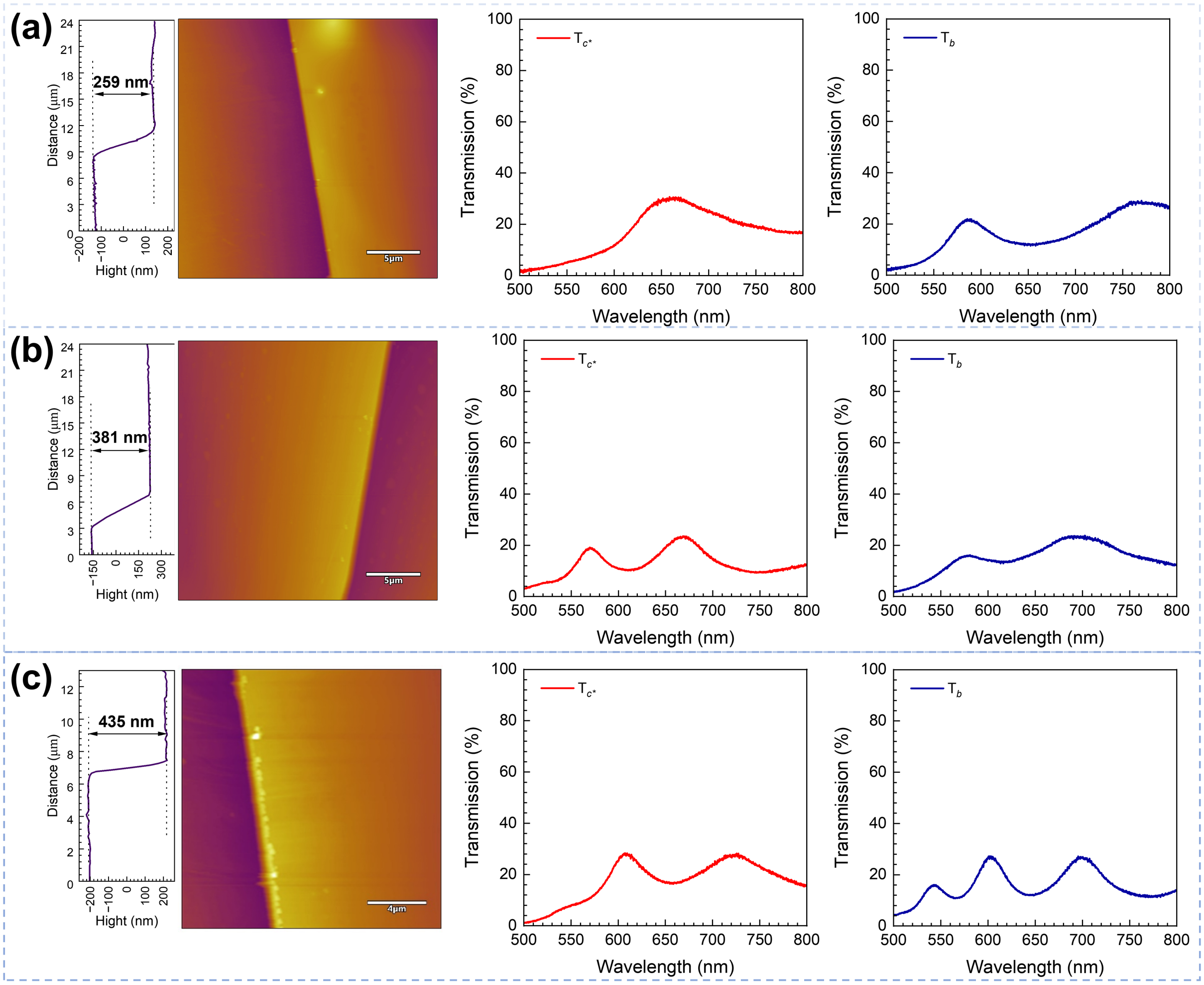

Supplementary Fig. 10 │ Thickness-dependent atomic force microscopy and polarized transmission of wavy-tube phosphorus crystals. Atomic force microscopy images and transmission spectra under polarization parallel and perpendicular to the *c**-axis for wavy-tube phosphorus (wtP) crystals with thicknesses of (a) 259 nm, (b) 381 nm, and (c) 435 nm , respectively.

As shown in Fig. S10, the transmission spectra of wtP samples at three different thicknesses (259 nm, 381 nm, and 435 nm) exhibit characteristic interference modulation. As sample thickness increases, the number of interference periods accommodated within the same spectral range grows, consistent with the physical principle of increased optical path difference. More significantly, the thickness-dependent interference effect markedly modulates the observed apparent anisotropy, where the relatively simple interference in the thinner 259 nm sample clearly reveals transmission

differences along and perpendicular to the c*-axis (Fig. S10a). However, as thickness increases to 381 nm and 435 nm, complex and asynchronous interference maxima and minima in both directions superimpose, producing a spectrally ‘smoothed’ effect that diminishes the contrast of the apparent anisotropy (Figs. S10b, c).

Conventionally, the optical properties of materials are extracted from reflectance spectra ($R(\omega)=r_0^2(\omega)$) combined with the Kramers-Kronig (K-K) relations. In such method, the phase shift $\theta(\omega_0)$ is derived to reconstruct the complex refractive index using the integral:

$$\theta(\omega_0)=-\frac{\omega_0}{\pi}P\int_0^{\infty}\frac{\ln R(\omega_0)}{\omega^2-\omega_0^2}d\omega$$

where ω is the angular frequency, and P denotes the Cauchy principal value. And the material’s optical constants yielding:

$$n(\omega)=\frac{1-r_0^2(\omega)}{1+r_0^2(\omega)-2r_0(\omega)\cos\theta(\omega)}$$
$$\kappa(\omega)=\frac{2r_0(\omega)\sin\theta(\omega)}{1+r_0^2(\omega)-2r_0(\omega)\cos\theta(\omega)}$$

However, this approach faces significant challenges when applied to thin films due to the Fabry-Pérot interference effect, governed by the condition:

$$2nd\cos\phi=m\lambda$$

where n is the refractive index, d is the film thickness, ϕ is the angle of refraction, λ is the wavelength, and m is an integer representing the interference order. In transparent or weakly absorbing regions, this interference can cause reflectance to fluctuate drastically between 10% and 90%, depending on the refractive index contrast. Consequently, applying K-K analysis introduces significant artifacts, with errors in the extracted absorption coefficient (α), and deviations in the refractive index (n).

In contrast, spectroscopic ellipsometry avoids these limitations by measuring the complex reflectance ratio ρ, defined as:

$$\rho = \frac{r_p}{r_s} = \tan(\Psi)\, e^{i\Delta}$$

Here, $r_p$and r_s are the complex reflection coefficients for parallel and perpendicular polarizations, respectively. By simultaneously measuring two independent parameters—the amplitude ratio (Ψ) and the phase difference (Δ)—this technique allows for the construction of an optical model where the film thickness d is treated as an independent fitting variable. This capability effectively decouples geometric interference effects from the intrinsic dielectric functions, yielding accurate optical constants.

As a result, we use spectroscopic ellipsometry in this work to determine optical parameters.

Section 7. Transmitted image of cross-polarized light

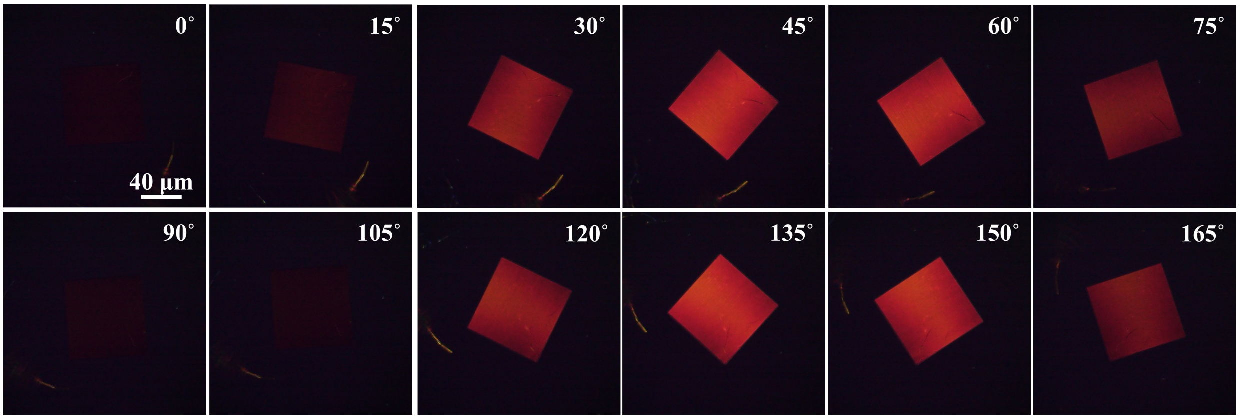

Supplementary Fig. 11 | Transmission images of wavy-tube phosphorus. Transmission images of wavy-tube phosphorus (wtP) crystals in cross-polarized light.

The wtP transmission images at various rotation angles are captured by a charge-coupled device (CCD) camera with an analyzer perpendicular to the incident polarized light. Initially, minimal transmitted light is detected, resulting in a dark image. As the incident polarized light passes through the crystals, it transforms into elliptically polarized light due to the birefringence effect. Consequently, the transmitted light is not entirely suppressed by the analyzer, leading to a bright image. Rotating the wtP crystal causes the image to exhibit periodic changes, with brightness depending on the sample position relative to the incident polarized light. When the incident light aligns with either the fast or slow axis of the crystals and the polarization state is maintained, the optical image appears darkest. Conversely, when the incident light is oriented at a 45° angle to the slow or fast axis, it causes the most significant change in the polarization state and the optical image appears brightest. Thus, polarization-resolved optical microscopy measurements clearly demonstrate the in-plane anisotropic refraction of wtP.

Section 8. Polar plot of Raman spectroscopy

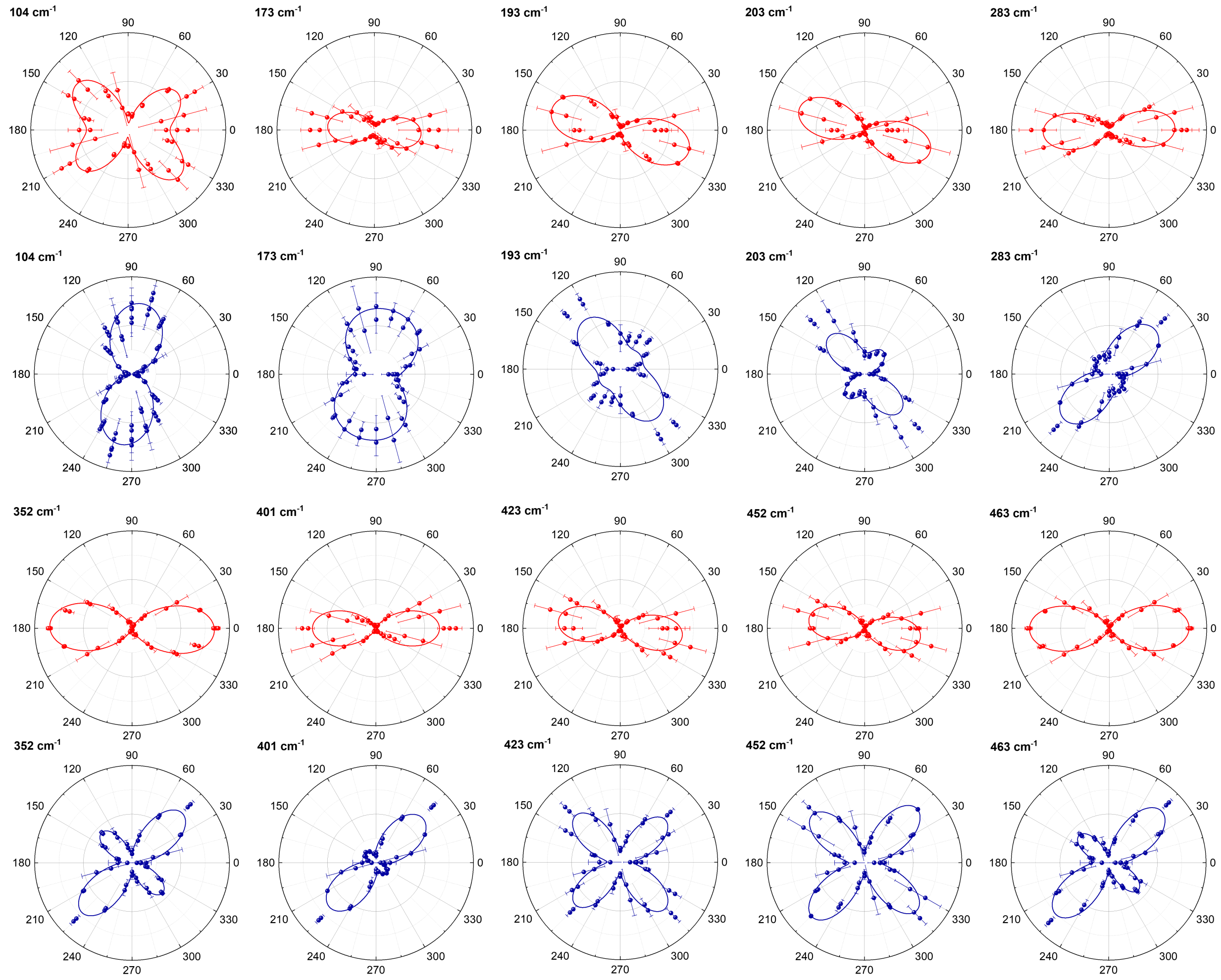

Supplementary Fig. 12 │ Polarized Raman polar plots of wavy-tube phosphorus crystal. Polar plots of different characteristic Raman peaks of the wavy-tube phosphorus (wtP) crystal in the parallel-polarized (red dots) and cross-polarized (blue dots) configurations. The signals are excited by a 633 nm laser. The fitted curves are in good agreement with the experimental data.

The angle-resolved polarized Raman spectra show that all the phonon modes exhibit strong anisotropy, with two- and four-lobed shapes in the co-polarized and cross-polarized configurations, respectively. Raman scattering is employed to quantify the polarization-dependent intensity, which is described by the following equation:[31] $I \propto \left|e_i \cdot R_j \cdot e_s\right|^2$, where e_i and e_s are the unit polarization

vectors of the incident laser and Raman signals, respectively. The wtP crystal has a monoclinic structure and belongs to the *C*2 point group (space group $P2_1$). According to group theory, the Raman tensor is described as[32,33]:

$$\mathrm{R}(A_g) = \begin{pmatrix} ae^{i\emptyset_a} & 0 & de^{i\emptyset_d} \\ 0 & be^{i\emptyset_b} & 0 \\ de^{i\emptyset_d} & 0 & ce^{i\emptyset_c} \end{pmatrix}.$$

Considering the absorbing Raman tensor, all the angular correlations are fitted by introducing the phase difference ϕ_{ab}. The anisotropic Raman scattering intensity in the co-polarized and cross-polarized configurations can be described as:

$$\mathrm{I}(A_g, \parallel) = a^2 \cos^4\theta + b^2 \sin^4\theta + 2ab \sin^2\theta \cos^2\theta \cos\emptyset_{ab} \quad \text{and} \tag{3}$$

$$\mathrm{I}(A_g, \perp) = a^2 \sin^2\theta \cos^2\theta + b^2 \sin^2\theta \cos^2\theta - 2ab \sin^2\theta \cos^2\theta \cos\emptyset_{ab}. \tag{4}$$

Section 9. Additional information for second-harmonic generation spectra

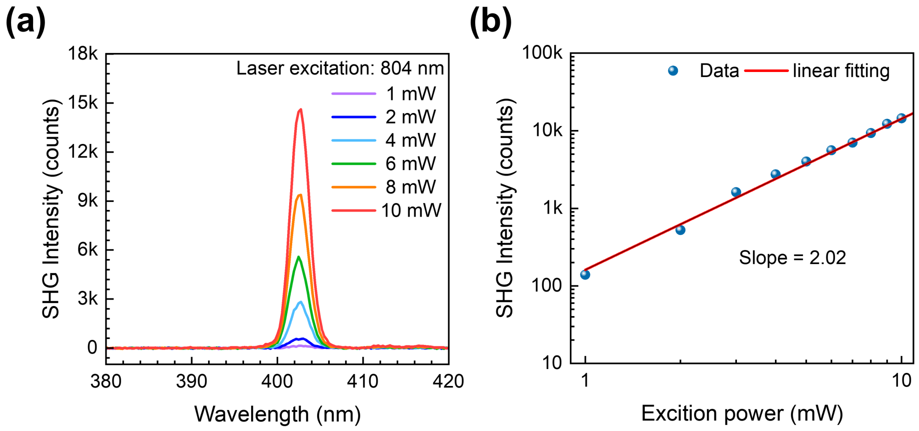

Supplementary Fig. 13 │ Power-dependent second-harmonic generation of wavy-tube phosphorus crystal. (a) Power-dependent second-harmonic generation (SHG) spectra of the wavy-tube phosphorus (wtP) crystal. (b) Fitted relationship between the SHG intensity and the excitation light power.

Supplementary Table 4 │ Comparison of the second-order susceptibility $\chi^{(2)}$ of wavy-tube phosphorus (wtP) with that of various other typical second-harmonic generation (SHG) materials.

**Material**	**$\chi^{(2)}$ (10^{-11} m/V)**	**References**
**wtP**	**1.95**	**This work**
Quartz	0.08	[34]
GaSe	1.8/1.1	[35,36]
$MoTe_2$	250	[37]
WS_2	0.2	[36]
InSe	1.3	[36]
$SnPS_3$	8.41	[38]
SnP_2S_6	406	[39]
$PdSe_2$	5.17	[40]
$LiNbO_3$	5.2	[41]

According to the following equation:[38] $\chi^{(2)} = \frac{\varepsilon_0^{1/2} c^{1/2} \lambda A^{1/2}}{8^{1/2}\pi} \cdot \frac{1}{P_\omega} \cdot \frac{1}{d} \cdot P_{2\omega}^{1/2} \cdot n_\omega n_{2\omega}^{1/2}$, where ε_0, c, A, and d are the vacuum dielectric constant, the speed of light, the incident light spot area, and the sample thickness, respectively. P_ω ($P_{2\omega}$) and n_ω ($n_{2\omega}$) are the laser (SHG) power and the refractive index at the fundamental (infra) frequency, respectively. The effective SHG susceptibility $\chi^{(2)}$ derived by the above equations in the wtP is ~1.95×10^{-11} m V^{-1}, which is comparable to that of many reported nonlinear materials (Table 4)[34,39].

Section 10. Energy band structure information obtained from ultraviolet photoelectron spectroscopy

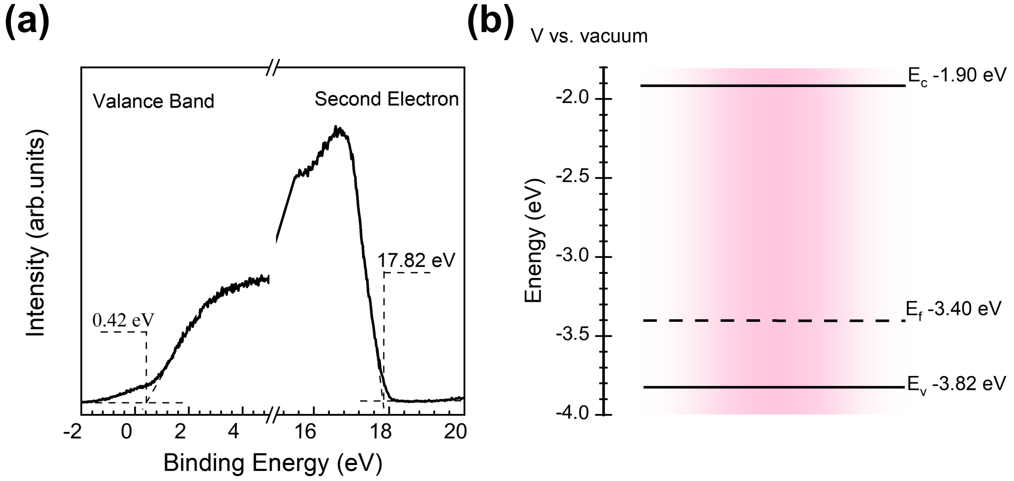

Supplementary Fig. 14 │ Work function and band edges of wavy-tube phosphorus crystal. (a) Work function and valence band edge of the bulk wavy-tube phosphorus (wtP) crystal. (b) Positions of the conductive band bottom (E_{CB}) and valent band top (E_{VB}) of bulk wtP on the absolute vacuum energy scale. The bulk wtP crystal energy bandgap of approximately 1.92 eV is measured by PL peak position (Fig. 4e). In the ultraviolet photoelectron spectroscopy (UPS) spectrum, the work function of the bulk wtP crystal is 3.4 eV by subtracting the secondary electron cut-off energy from the optical energy of the He I source (21.22 eV), and the valence band top to Fermi energy level of the bulk wtP crystal is about 0.42 eV. Therefore, the conduction band bottom of the bulk wtP crystal is calculated to be about -1.90 eV by the $E_{BG} = E_{VB} + E_{CB}$ equation.

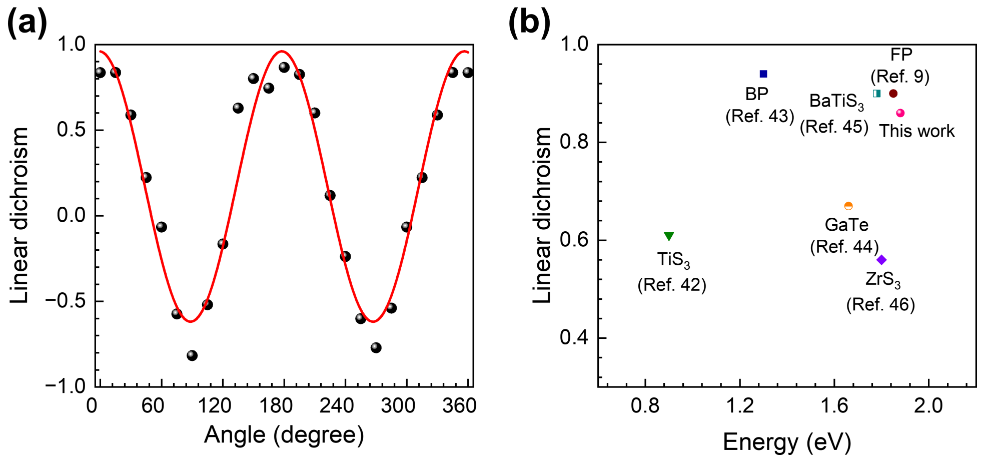

Supplementary Fig. 15 │ Photoluminescence linear dichroism of wavy-tube phosphorus crystal. (a) Linear dichroism of photoluminescence (PL) peak intensity versus polarization angle θ. (b) Comparison of the PL emission linear dichroism of wavy-tube phosphorus (wtP) with other low-dimensional materials[9,42-46], in which the horizontal axis indicates the PL wavelength corresponded energy.

The linear dichroism is defined as: Linear dichroism $=\frac{\mathrm{I_{co}}-\mathrm{I_{cross}}}{\mathrm{I_{co}}+\mathrm{I_{cross}}}$, where I_{co} and I_{cross} denote the photoluminescence emission in two crystal directions orthogonal to each other.

Supplementary References:

1. Bridgman, P. Two new modifications of phosphorus. *J. Am. Chem. Soc.* **36**, 1344-1363 (1914).
2. Brown, A. & Rundqvist, S. Refinement of the crystal structure of black phosphorus. *Acta Crystallogr.* **19**, 684-685 (1965).
3. Xia, F., Wang, H. & Jia, Y. Rediscovering black phosphorus as an anisotropic layered material for optoelectronics and electronics. *Nat. Commun.* **5**, 4458 (2014).
4. Roth, W. L., De, W. T. & Smith, A. J. Polymorphism of red phosphorus. *J. Am. Chem. Soc.* **69**, 2881-2885 (1947).
5. Hittorf, W. Zur kenntniss des phosphors. *Ann. Phys.* **202**, 193-228 (1865).
6. Zhang, L. *et al.* Structure and Properties of Violet Phosphorus and Its Phosphorene Exfoliation. *Angew. Chem. Int. Ed.* **59**, 1074-1080 (2020).
7. Thurn, H. & Krebs, H. Über struktur und eigenschaften der halbmetalle. XXII. Die kristallstruktur des hittorfschen phosphors. *Acta Cryst. B* **25**, 125-135 (1969).
8. Ruck, M. *et al.* Fibrous red phosphorus. *Angew. Chem. Int. Ed.* **44**, 7616-7619 (2005).
9. Du, L. *et al.* Giant anisotropic photonics in the 1D van der Waals semiconductor fibrous red phosphorus. *Nat. Commun.* **12**, 4822 (2021).
10. Shao, J. *et al.* Biodegradable black phosphorus-based nanospheres for in vivo photothermal cancer therapy. *Nat. Commun.* **7**, 12967 (2016).
11. Carvalho, A. *et al.* Phosphorene: from theory to applications. *Nat. Rev. Mater.* **1** (2016).
12. Ricciardulli, A. G., Wang, Y., Yang, S. & Samori, P. Two-Dimensional Violet Phosphorus: A p-Type Semiconductor for (Opto)electronics. *J. Am. Chem. Soc.* **144**, 3660-3666 (2022).
13. Wu, C. *et al.* Hetero-phase dendritic elemental phosphorus for visible light photocatalytic hydrogen generation. *Appl. Catal. B: Environ.* **312**, 121428 (2022).
14. Liu, Y., Hu, Z. & Yu, J. C. Liquid bismuth initiated growth of phosphorus microbelts with efficient charge polarization for photocatalysis. *Appl. Catal. B: Environ.* **247**, 100-106 (2019).
15. Winchester, R. A., Whitby, M. & Shaffer, M. S. Synthesis of pure phosphorus nanostructures. *Angew. Chem. Int. Ed.* **48**, 3616-3621 (2009).
16. Sun, Z., Zhang, B. & Yan, Q. Solution phase synthesis of the less-known Form II crystalline red phosphorus. *Inorg. Chem. Front.* **9**, 4385-4393 (2022).
17. Yoon, J. Y. *et al.* Type-II Red Phosphorus: Wavy Packing of Twisted Pentagonal Tubes. *Angew. Chem. Int. Ed.* **62**, e202307102 (2023).
18. Qiu, P. *et al.* Photo-Assisted Bottom-Up Synthesis of Orange Phosphorus. *Angew. Chem. Int. Ed.* **64**, e202421571 (2025).
19. Zhang, B. *et al.* Assessing the Structural Diversity of Form II Red Phosphorus via Stepwise Crystal Structure Search. *J. Am. Chem. Soc.* **146**, 26369-26378 (2024).
20. Rubenstein, M. & F. M. Ryan. Allotropes of red phosphorus. *J. Electrochem. Soc.* **113**, 1063-1067 (1966).
21. Schäfer, H. & M. Trenkel. Catalyzed sublimation of red phosphorus. *Z. Anorg. Allg. Chem.* **391**, 11-18 (1972).
22. Duan, Z. *et al.* Large-Scale synthesis of crystalline phosphorus nanosheets with superior air-water stability and flame-retardancy ability. *Chem. Eng. J.* **505**, 159566 (2025).
23. Pfitzner, A. Phosphorus remains exciting! *Angew. Chem. Int. Ed.* **45**, 699-700 (2006).
24. Jamieson, J. C. Crystal structures adopted by black phosphorus at high pressures. *Science* **139**, 1291-1292 (1963).

25. Liu, Q. *et al.* Crystalline Red Phosphorus Nanoribbons: Large-Scale Synthesis and Electrochemical Nitrogen Fixation. *Angew. Chem. Int. Ed.* **59**, 14383-14387 (2020).
26. Zhang, S. *et al.* Synthesis of Fibrous Phosphorus Micropillar Arrays with Pyro-Phototronic Effects. *Angew. Chem. Int. Ed.* **62**, e202217127 (2023).
27. Zhang, S. *et al.* Controllable preparation of crystalline red phosphorus and its photocatalytic properties. *Nanoscale* **13**, 18955-18960 (2021).
28. Guo, Z. *et al.* Metal-Ion-Modified Black Phosphorus with Enhanced Stability and Transistor Performance. *Adv. Mater.* **29**, 1703811 (2017).
29. Zhang, S. *et al.* Crystallization kinetics of amorphous red phosphorus to black phosphorus by chemical vapor transport. *CrystEngComm* **24**, 504-511 (2022).
30. Chen, C. *et al.* Growth of single-crystal black phosphorus and its alloy films through sustained feedstock release. *Nat. Mater.* **22**, 717-724 (2023).
31. Zou, B. *et al.* Unambiguous determination of crystal orientation in black phosphorus by angle-resolved polarized Raman spectroscopy. *Nanoscale Horiz.* **6**, 809-818 (2021).
32. Loudon, R. The Raman effect in crystals. *Adv. Phys.* **13**, 423-482 (1964).
33. Ribeiro, H. B. *et al.* Unusual Angular Dependence of the Raman Response in Black Phosphorus. *ACS Nano* **9**, 4270-4276 (2015).
34. Manaka, T., Lim, E., Tamura, R. & Iwamoto, M. Direct imaging of carrier motion in organic transistors by optical second-harmonic generation. *Nat. Photonics* **1**, 581-584 (2007).
35. Zhou, X. *et al.* Strong Second-Harmonic Generation in Atomic Layered GaSe. *J. Am. Chem. Soc.* **137**, 7994-7997 (2015).
36. Hao, Q. *et al.* Phase Identification and Strong Second Harmonic Generation in Pure epsilon-InSe and Its Alloys. *Nano Lett.* **19**, 2634-2640 (2019).
37. Song, Y. *et al.* Second Harmonic Generation in Atomically Thin $MoTe_2$. *Adv. Opt. Mater.* **6**, 1701334 (2018).
38. Yang, Y. *et al.* A Universal Strategy for Synthesis of 2D Ternary Transition Metal Phosphorous Chalcogenides. *Adv. Mater.* **36**, e2307237 (2024).
39. Zhang, Y. *et al.* Inversion symmetry broken 2D SnP_2S_6 with strong nonlinear optical response. *Nano Res.* **15**, 2391-2398 (2021).
40. Yu, J. *et al.* Giant nonlinear optical activity in two-dimensional palladium diselenide. *Nat. Commun.* **12**, 1083 (2021).
41. Abdelwahab, I. *et al.* Giant second-harmonic generation in ferroelectric $NbOI_2$. *Nat. Photonics* **16**, 644-650 (2022).
42. Khatibi, A. *et al.* Anisotropic infrared light emission from quasi-1D layered TiS_3. *2D Mater.* **7**, 015022 (2019).
43. Wang, X. *et al.* Highly anisotropic and robust excitons in monolayer black phosphorus. *Nat. Nanotechnol.* **10**, 517-521 (2015).
44. Cai, H. *et al.* Synthesis of Highly Anisotropic Semiconducting GaTe Nanomaterials and Emerging Properties Enabled by Epitaxy. *Adv. Mater.* **29**, 1605551 (2017).
45. Wu, J. *et al.* Linear Dichroism Conversion in Quasi-1D Perovskite Chalcogenide. *Adv. Mater.* **31**, e1902118 (2019).
46. Pant, A. *et al.* Strong dichroic emission in the pseudo one dimensional material ZrS_3. *Nanoscale* **8**, 16259-16265 (2016).
