# Supplementary material for: Strong optical anisotropy in one-dimensional phosphorus wavy tubes
Source: Nat Commun. 2026 Feb 28;17:3286. doi: 10.1038/s41467-026-70129-4 (PMC13066532; doi:10.1038/s41467-026-70129-4)
Supplement: Supplementary file 2 — Description of Additional Supplementary Files [file 41467_2026_70129_MOESM2_ESM.pdf]

Description of Additional Supplementary Files

File Name: Supplementary Data 1
Description: cif file of wavy-tube phosphorus;

File Name: Supplementary Data 2
Description: hkl file of wavy-tube phosphorus;

File Name: Supplementary Data 3
Description: CheckCIF file of wavy-tube phosphorus;
