# Supplementary material for: Strong optical anisotropy in one-dimensional phosphorus wavy tubes
Source: Nat Commun. 2026 Feb 28;17:3286. doi: 10.1038/s41467-026-70129-4 (PMC13066532; doi:10.1038/s41467-026-70129-4)

checkCIF/PLATON report

You have not supplied any structure factors. As a result the full set of tests cannot be run.

THIS REPORT IS FOR GUIDANCE ONLY. IF USED AS PART OF A REVIEW PROCEDURE FOR PUBLICATION, IT SHOULD NOT REPLACE THE EXPERTISE OF AN EXPERIENCED CRYSTALLOGRAPHIC REFEREE.

No syntax errors found.   CIF dictionary   Interpreting this report

Datablock: p

```
Bond precision:   P- P = 0.0105 A          Wavelength=1.54184

Cell:            a=13.0518(3)     b=34.4922(4)        c=18.8538(4)
                 alpha=90         beta=109.737(3)     gamma=90
Temperature:     293 K

                Calculated                 Reported
Volume          7989.1(3)                  7989.1(3)
Space group     P 21                       P 1 21 1
Hall group      P 2yb                      P 2yb
Moiety formula  P60                        3(P0.333)
Sum formula     P60                        P
Mr              1858.20                    30.97
Dx,g cm-3       2.317                      2.317
Z               6                          360
Mu (mm-1)       17.484                     17.484
F000            5400.0                     5400.0
F000'           5508.34
h,k,lmax        16,43,23                   16,43,23
Nref            33590[ 17084]              26956
Tmin,Tmax       0.233,0.840                0.461,1.000
Tmin'           0.024

Correction method= # Reported T Limits: Tmin=0.461 Tmax=1.000
AbsCorr = MULTI-SCAN

Data completeness= 1.58/0.80       Theta(max)= 76.435

                                                    wR2(reflections)=
R(reflections)= 0.0858( 24131)                      0.2801( 26956)

S = 1.266                    Npar= 1624
```

The following ALERTS were generated. Each ALERT has the format
test-name_ALERT_alert-type_alert-level.
Click on the hyperlinks for more details of the test.

Alert level A

PLAT029_ALERT_3_A _diffrn_measured_fraction_theta_full value Low . 0.754 Why?

Alert level C

SHFSU01_ALERT_2_C The absolute value of parameter shift to su ratio > 0.05
Absolute value of the parameter shift to su ratio given 0.065
Additional refinement cycles may be required.
STRVA01_ALERT_2_C Chirality of atom sites is inverted?
From the CIF: _refine_ls_abs_structure_Flack 1.300
From the CIF: _refine_ls_abs_structure_Flack_su 0.700
PLAT042_ALERT_1_C Calc. and Reported MoietyFormula Strings Differ Please Check
Calc: P60
Rep.: 3(P0.333)
PLAT080_ALERT_2_C Maximum Shift/Error .... 0.06 Why ?
PLAT084_ALERT_3_C High wR2 Value (i.e. > 0.25) .... 0.28 Report
PLAT907_ALERT_2_C Flack x > 0.5, Structure Needs to be Inverted? . 1.30 Check

Alert level G

PLAT004_ALERT_5_G Polymeric Structure Found with Maximum Dimension 1 Info
PLAT012_ALERT_1_G No _shelx_res_checksum Found in CIF ...... Please Check
PLAT032_ALERT_4_G Std. Uncertainty on Flack Parameter Value High . 0.700 Report
PLAT045_ALERT_1_G Calculated and Reported Z Differ by a Factor ... 0.017 Check
PLAT072_ALERT_2_G SHELXL First Parameter in WGHT Unusually Large 0.20 Report
PLAT199_ALERT_1_G Reported _cell_measurement_temperature ..... (K) 293 Check
PLAT200_ALERT_1_G Reported _diffrn_ambient_temperature ..... (K) 293 Check
PLAT650_ALERT_4_G SWAT Instruction Used to Model Solvent Disorder ! Report
PLAT720_ALERT_4_G Number of Unusual/Non-Standard Labels .... 180 Note

P001	P002	P003	P004	P005	P006	P007	P008
P009	P00A	P00B	P00C	P00D	P00E	P00F	P00G
P00H	P00I	P00J	P00K	P00L	P00M	P00N	P00O
P00P	P00Q	P00R	P00S	P00T	P00U	P00V	P00W
P00X	P00Y	P00Z	P010	P011	P012	P013	P014
P015	P016	P017	P018	P019	P01A	P01B	P01C
P01D	P01E	P01F	P01G	P01H	P01I	P01J	P01K
P01L	P01M	P01N	P01O	P01P	P01Q	P01R	P01S
P01T	P01U	P01V	P01W	P01X	P01Y	P01Z	P020
P021	P022	P023	P024	P025	P026	P027	P028
P029	P02A	P02B	P02C	P02D	P02E	P02F	P02G
P02H	P02I	P02J	P02K	P02L	P02M	P02N	P02O
P02P	P02Q	P02R	P02S	P02T	P02U	P02V	P02W
P02X	P02Y	P02Z	P030	P031	P032	P033	P034
P035	P036	P037	P038	P039	P03A	P03B	P03C
P03D	P03E	P03F	P03G	P03H	P03I	P03J	P03K
P03L	P03M	P03N	P03O	P03P	P03Q	P03R	P03S
P03T	P03U	P03V	P03W	P03X	P03Y	P03Z	P040
P041	P042	P043	P044	P045	P046	P047	P048
P049	P04A	P04B	P04C	P04D	P04E	P04F	P04G
P04H	P04I	P04J	P04K	P04L	P04M	P04N	P04O

```
              P04P    P04Q    P04R    P04S    P04T    P04U    P04V    P04W
              P04X    P04Y    P04Z    P050
PLAT804_ALERT_5_G Number of ARU-Code Packing Problem(s) in PLATON          36 Info
PLAT933_ALERT_2_G Number of HKL-OMIT Records in Embedded .res File          2 Note
              -15  3 10,  15  2  0,
```

```
  1 ALERT level A = Most likely a serious problem - resolve or explain
  0 ALERT level B = A potentially serious problem, consider carefully
  6 ALERT level C = Check. Ensure it is not caused by an omission or oversight
 11 ALERT level G = General information/check it is not something unexpected

  5 ALERT type 1 CIF construction/syntax error, inconsistent or missing data
  6 ALERT type 2 Indicator that the structure model may be wrong or deficient
  2 ALERT type 3 Indicator that the structure quality may be low
  3 ALERT type 4 Improvement, methodology, query or suggestion
  2 ALERT type 5 Informative message, check
```

It is advisable to attempt to resolve as many as possible of the alerts in all categories. Often the minor alerts point to easily fixed oversights, errors and omissions in your CIF or refinement strategy, so attention to these fine details can be worthwhile. In order to resolve some of the more serious problems it may be necessary to carry out additional measurements or structure refinements. However, the purpose of your study may justify the reported deviations and the more serious of these should normally be commented upon in the discussion or experimental section of a paper or in the "special_details" fields of the CIF. checkCIF was carefully designed to identify outliers and unusual parameters, but every test has its limitations and alerts that are not important in a particular case may appear. Conversely, the absence of alerts does not guarantee there are no aspects of the results needing attention. It is up to the individual to critically assess their own results and, if necessary, seek expert advice.

Publication of your CIF in IUCr journals

A basic structural check has been run on your CIF. These basic checks will be run on all CIFs submitted for publication in IUCr journals (*Acta Crystallographica*, *Journal of Applied Crystallography*, *Journal of Synchrotron Radiation*); however, if you intend to submit to *Acta Crystallographica Section C* or *E* or *IUCrData*, you should make sure that full publication checks are run on the final version of your CIF prior to submission.

Publication of your CIF in other journals

Please refer to the *Notes for Authors* of the relevant journal for any special instructions relating to CIF submission.

Validation response form

Please find below a validation response form (VRF) that can be filled in and pasted into your CIF.

```
# start Validation Reply Form
_vrf_SHFSU01_p
;
PROBLEM: The absolute value of parameter shift to su ratio > 0.05
RESPONSE: ...
;
_vrf_STRVA01_p
;
PROBLEM: Chirality of atom sites is inverted?
RESPONSE: ...
;
_vrf_PLAT029_p
;
PROBLEM: _diffrn_measured_fraction_theta_full value Low .      0.754 Why?
RESPONSE: ...
;
_vrf_PLAT042_p
;
PROBLEM: Calc. and Reported MoietyFormula Strings  Differ     Please Check
RESPONSE: ...
;
_vrf_PLAT080_p
;
PROBLEM: Maximum Shift/Error ...........................       0.06 Why ?
RESPONSE: ...
;
_vrf_PLAT084_p
;
PROBLEM: High wR2 Value (i.e. > 0.25) ..................       0.28 Report
RESPONSE: ...
;
_vrf_PLAT907_p
;
PROBLEM: Flack x > 0.5, Structure Needs to be Inverted? .       1.30 Check
RESPONSE: ...
;
# end Validation Reply Form
```

PLATON version of 13/12/2023; check.def file version of 13/12/2023

Datablock p - ellipsoid plot

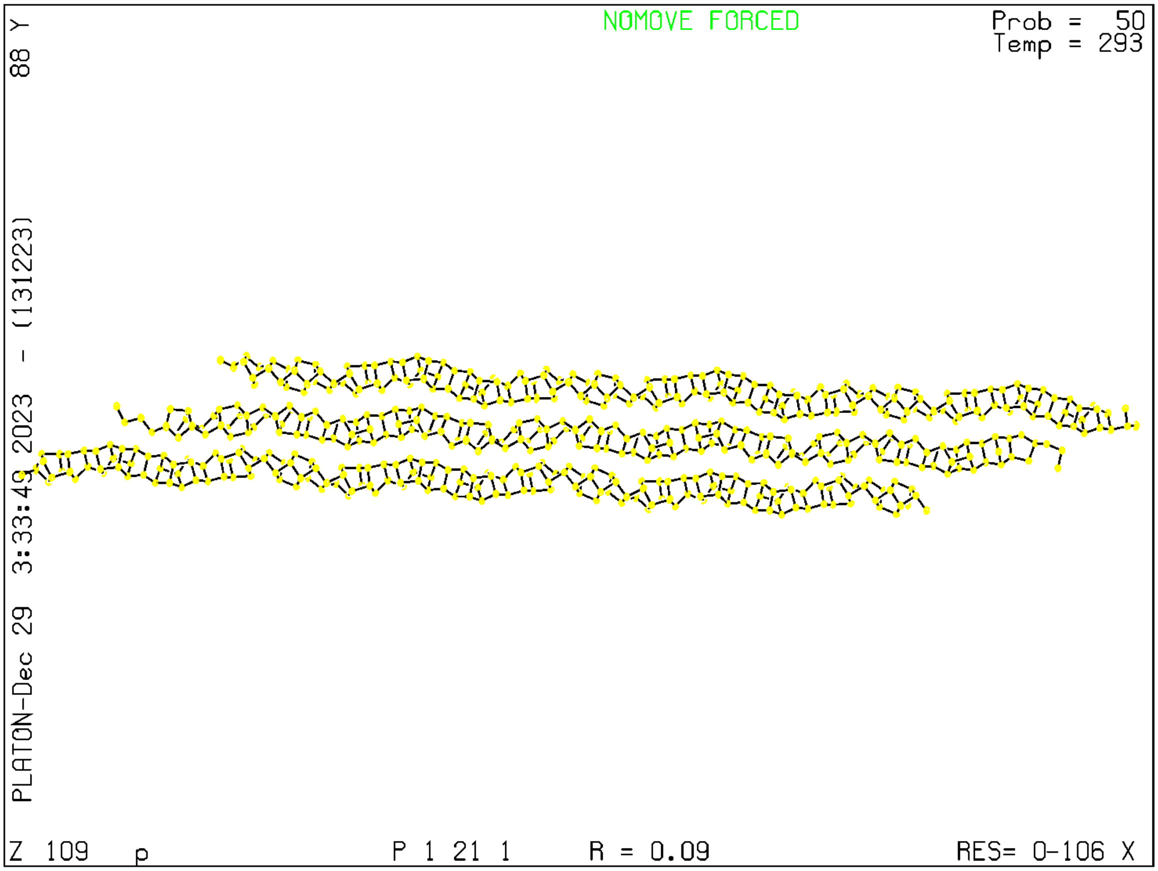

Supplement: Supplementary file 5 — Supplementary Data 3 [file 41467_2026_70129_MOESM5_ESM.pdf]
